# Crystal Structure and Concentration-Driven Phase Transitions in Lu_(1−*x*)_Sc*_x_*FeO_3_ (0 ≤ *x* ≤ 1) Prepared by the Sol–Gel Method

**DOI:** 10.3390/ma15031048

**Published:** 2022-01-29

**Authors:** Andrius Pakalniškis, Denis O. Alikin, Anton P. Turygin, Alexander L. Zhaludkevich, Maxim V. Silibin, Dmitry V. Zhaludkevich, Gediminas Niaura, Aleksej Zarkov, Ramūnas Skaudžius, Dmitry V. Karpinsky, Aivaras Kareiva

**Affiliations:** 1 Institute of Chemistry, Vilnius University, Naugarduko 24, LT-03225 Vilnius, Lithuania; ramunas.skaudzius@chgf.vu.lt (R.S.); aivaras.kareiva@chgf.vu.lt (A.K.); 2 School of Natural Sciences and Mathematics, Ural Federal University, 620000 Ekaterinburg, Russia; denis.alikin@ua.pt (D.O.A.); anton.turygin@urfu.ru (A.P.T.); 3 Department of Physics & CICECO—Aveiro Institute of Materials, University of Aveiro, 3810-193 Aveiro, Portugal; 4 Scientific-Practical Materials Research Centre of NAS of Belarus, 220072 Minsk, Belarus; zheludkevich27@gmail.com (A.L.Z.); geludkevichdima@mail.ru (D.V.Z.); dmitry.karpinsky@gmail.com (D.V.K.); 5 Institute of Advanced Materials and Technologies, National Research University of Electronic Technology “MIET”, 124498 Moscow, Russia; sil_m@mail.ru; 6 Institute for Bionic Technologies and Engineering, I.M. Sechenov First Moscow State Medical University, 119991 Moscow, Russia; 7 Department of Organic Chemistry, Center for Physical Sciences and Technology (FTMC), Sauletekio Ave. 3, LT-10257 Vilnius, Lithuania; gediminas.niaura@ftmc.lt; 8 Department of Materials Science and Physical Chemistry of Materials, South Ural State University, Av. Lenina, 76, 454080 Chelyabinsk, Russia

**Keywords:** phase transitions, structural phase stability, LuFeO_3_, X-ray diffraction, solid solutions

## Abstract

The structural state and crystal structure of Lu_(1−*x*)_Sc*_x_*FeO_3_ (0 ≤ *x* ≤ 1) compounds prepared by a chemical route based on a modified sol–gel method were investigated using X-ray diffraction, Raman spectroscopy, as well as scanning electron microscopy. It was observed that chemical doping with Sc ions led to a structural phase transition from the orthorhombic structure to the hexagonal structure via a wide two-phase concentration region of 0.1 < *x* < 0.45. An increase in scandium content above 80 mole% led to the stabilization of the non-perovskite bixbyite phase specific for the compound ScFeO_3_. The concentration stability of the different structural phases, as well as grain morphology, were studied depending on the chemical composition and synthesis conditions. Based on the data obtained for the analyzed samples, a composition-dependent phase diagram was constructed.

## 1. Introduction

The crystal structure and properties of compounds with perovskite structure (nominal chemical formula ABO_3_) [1,2,3,4] can be drastically modified by a chemical substitution in A- and/or B-perovskite sublattices. The introduction of elements of different ionic radii leads to the stabilization of diverse types of structural distortion described by the Goldschmidt tolerance factor [5,6,7]. The possibility to control physical properties via chemical doping is particularly important in regard to the formation of both electrical and magnetic orderings in these compounds, which are commonly referred to as multiferroics [8,9]. These conditions are often contradictive, since magnetic ordering usually requires partially filled d orbitals, whereas electrical ordering requires empty d orbitals of the ions occupying the B-perovskite position. However, nowadays different mechanisms allowing ferroelectricity have been discovered (lone pair, charge ordering, geometric, and spin-driven) that permit the coexistence of both types of ordering [9,10]. The formation of efficient multiferroic compounds allows controlling their electric properties by a magnetic field and vice versa, making these compounds potentially useful for various practical applications [8,11,12,13]. 

Probably, BiFeO_3_ is the most investigated and common multiferroic compound; however, this compound suffers from a large leakage current and is difficult to prepare. Therefore, alternatives are needed [14]. Recently, a new family of room-temperature multiferroic compounds based on LuFeO_3_ with hexagonal structure (space group *P6*_3_*cm*) has been discovered [15,16]. It was found that LuFeO_3_ in the hexagonal state has both ferroelectric and weak ferromagnetic ordering [17,18,19]. Furthermore, it has been reported that the compound in orthorhombic phase (space group *Pnma*) is antiferromagnetic below 620 K, while being in hexagonal structure, the magnetic transition shifts down to 440 K, and below 130 K, the compound has weak ferromagnetism, due to canting towards the c-axis, with the polarization being retained up to 1050 K, at least in the case of thin films [18,20]. The crystal structure and the origin of multiferroicity are similar to those specific for hexagonal manganites (e.g., YMnO_3_) [21].

It should be noted that the preparation of hexagonal compounds is quite difficult, and the crystal structure can be realized either using chemical substitution or in the form of thin films, as lattice is unstable under external stimuli and tends to form an orthorhombic structure specific for orthoferrites [22,23,24,25]. Due to the aforementioned unstable nature of the lattice and the difficulty of preparation and characterization of the hexagonal variant of LuFeO_3_, the main available results regard thin films [16,17,18]. However, when analyzing thin films, it is important to take into account the effect of strain and interface interactions, as they can significantly affect thin films’ chemical and physical properties [15,21]. Thus, in order to fully analyze the system, it is important to perform the experiments on bulk samples of pure chemical composition. Recently, it has been shown that it is possible to stabilize the hexagonal structure of LuFeO_3_ using chemical doping of Lu ions by other ions with similar ionic radii (e.g., Sc, Yb, Ho) using solid-state methods [26,27]. Additionally, similar structural changes can be induced by changing the cation in B position; in this case, Fe^3+^ can be substituted with another transition metal ion, e.g., Ni and Mn [28,29]. Alternatively, the crystal structure of the parent compound can be affected by the formation of LuFeO_3_-based compounds using doping either by other perovskite compounds, e.g., YMnO_3_ [30], or via diffusion into the perovskite matrix of pure iron [31].

However, there is contradictory information in the literature [26,27,29] describing the crystal structure of these compounds and their structural stability under internal (grain size, morphology, local stresses, synthesis conditions, etc.) and external (temperature, pressure, electric, and magnetic fields) stimuli. Thus, additional and detailed investigations are required. On the other hand, recently, ferroelectricity was demonstrated in single crystals of LuFeO_3_ doped by Sc, which makes Sc a promising candidate to prepare bulk ferroelectric polycrystalline samples with interesting potential applications [32].

Taking the mentioned arguments into account, in this paper, we provide a new route to prepare Sc-doped LuFeO_3_ polycrystalline compounds using an aqueous sol–gel synthesis procedure and provide clarification of the concentration ranges of the different structural phases present in the system and analyzed by means of SEM, EDX/EDS, X-ray diffraction, and Raman spectroscopy. The obtained results allowed determining the evolution of crystal symmetry, structural parameters, and crystalline morphology depending on the annealing conditions using the mentioned preparation method as well as analyzing this information in relation to the available structural data. Furthermore, a relation between phase formation and particle morphology was also established.

## 2. Materials and Methods

Lu_2_O_3_ (99.99%, Sigma Aldrich, St. Louis, MO, USA), Sc_2_O_3_ (99.9%, Sigma Aldrich), Fe(NO_3_)_3_⋅9H_2_O (98%, Alfa Aesar, Haverhill, MA, USA), ethylene glycol (EG, Lach-ner, Neratovice, Czechia), and concentrated HNO_3_ (Lach-ner) were used as precursors. Samples containing 0, 0.15, 0.25, 0.50, 0.75, 0.80, and 1 Sc were prepared. For the preparation of the samples, ethylene glycol-assisted sol–gel synthesis was used, obtaining for each nominal synthesis, about 1 g of the final product. Initially, Sc_2_O_3_ was dissolved in 25 mL of HNO_3_ at around 100 °C (temperature of the magnetic stirrer) in a covered chemical glass under constant magnetic stirring. This was followed by the addition of Lu_2_O_3_, which was also dissolved in the solution. After both oxides had dissolved, nitric acid was evaporated, and the remaining liquid was washed with distilled water and finally diluted to a volume of 50 mL. Subsequently, iron nitrate was added, and the obtained solution was stirred for 1 h at around 80 °C. Then, ethylene glycol was added in a ratio of 1:1 to metal cations, and the solution was further stirred for 1 h to obtain a homogeneous solution. Finally, the solution was evaporated at 200 °C, until a gel was obtained, which was dried overnight in a furnace at 150 °C. The obtained xerogel was ground in an agate mortar and calcinated at the temperatures of 500, 650, 800, 950, 1100 °C for 1.5 h with a heating rate of 1 °C/min. Samples calcinated at 1100 °C (with 0.00, 0.25, 0.50, 0.75, 1.00 of Sc) were further sintered at 1300 and 1500 °C for 3 h at a heating rate of 5 °C/min.

XRD measurements were performed using a Rigaku MiniFlex II diffractometer using Cu Kα radiation (λ = 1.5418 Å) in 2*θ* range from 10° to 80° with a scan speed of 5°/min and 0.02° step size. The measurement current was set to 15 mA, and the voltage was set to 30 kV. Structural Rietveld refinement was performed using FullProf software [33]. The dual-beam system FE-SEM-FIB Helios Nanolab 650 with an energy-dispersive X-ray (EDX) spectrometer INCA Energy 350 with an X-Max 20 SDD detector was employed for the measurement of chemical composition as well as for the preparation of SEM micrographs. Raman scattering measurements were conducted using an inVia Raman (Renishaw, United Kingdom) spectrometer equipped with a thermoelectrically cooled (−70 °C) CCD camera and a microscope. Raman spectra were obtained by excitation with a 532 nm beam from the CW diode-pumped solid-state (DPSS) laser (Renishaw, UK). To avoid damage of the sample, the laser power at the sample was restricted to a very low value, 0.06 mW. A 20×/0.40 NA objective lens and 1800 lines/mm grating were used during all measurements. The overall integration time was 800 s. The position of the Raman bands on the wavenumber axis was calibrated by the polystyrene film standard spectrum. The parameters of the bands were determined by fitting the experimental spectra with Gaussian–Lorentzian shape components using GRAMS/A1 8.0 (Thermo Scientific, Waltham, MA, USA) software. 

## 3. Results

### 3.1. Crystal Structure of the Compounds by Diffraction Measurements

The diffraction data obtained for LuFeO_3_ (Figure S1) indicated the formation of an amorphous phase in the xerogels sintered at temperatures up to 650 °C. However, further increase in the temperature up to 800 °C led to the formation of a typical orthoferrite phase with orthorhombic symmetry, described by the *Pnma* (#62) space group [25]. An additional increase in temperature up to 950 and 1100 °C led to a narrowing of the diffraction peaks and a notable increase in their intensity, which was caused by increased crystallinity and crystallite size. A slightly different behavior was found for the ScFeO_3_ samples. Even at the initial sintering temperature of 650 °C, multiple diffraction peaks were observed, indicating the formation of a crystalline phase (Figure S2). This phase is consistent with the formation of a bixbyite-type structure of ScFeO_3_ having cubic symmetry and described by the space group *Ia-3* (#206); a small amount of an unidentified impurity phase was also observed. The mixed structural state was consistent up to 950 °C; however, the reflections specific for the bixbyite structure notably increased in intensity, relatively to the impurity phase. At the temperature of 1100 °C, a single-phase cubic bixbyite structure formed, but even in this case, the diffraction pattern was characterized by a quite high-intensity background, indicating a residual amorphous component and the need of an even higher sintering temperature. Furthermore, it is worthy to note that the bixbyite structure could no longer be considered a perovskite, since in the structure, the Sc and Fe ions were distributed randomly due to their relatively similar ionic radii.

**Figure 1:**
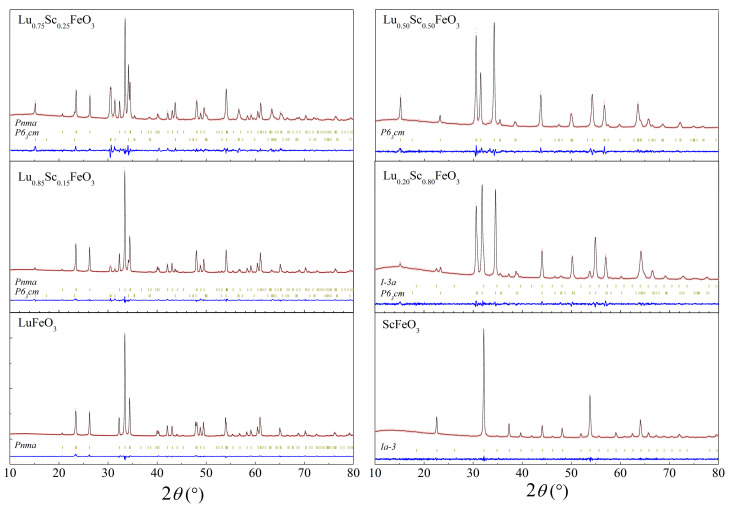
Rietveld refinement results of XRD data recorded for Lu_(1−*x*)_Sc*_x_*FeO_3_ (0 ≤ *x* ≤ 1.00) compounds at room temperature (red dots are the experimental data; black lines are calculated data), Bragg reflections are indicated by vertical ticks.

**Figure 2:**
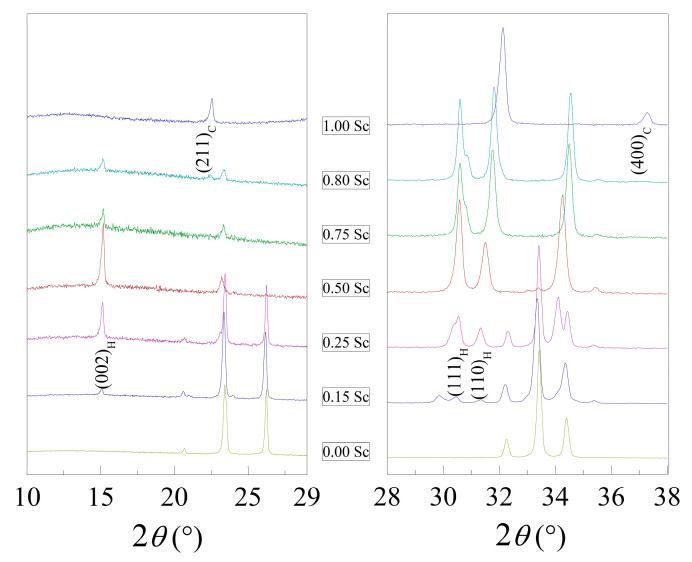
Phase specific peaks for *P6_3_cm* and *Ia-3* space groups.

**Figure 3:**
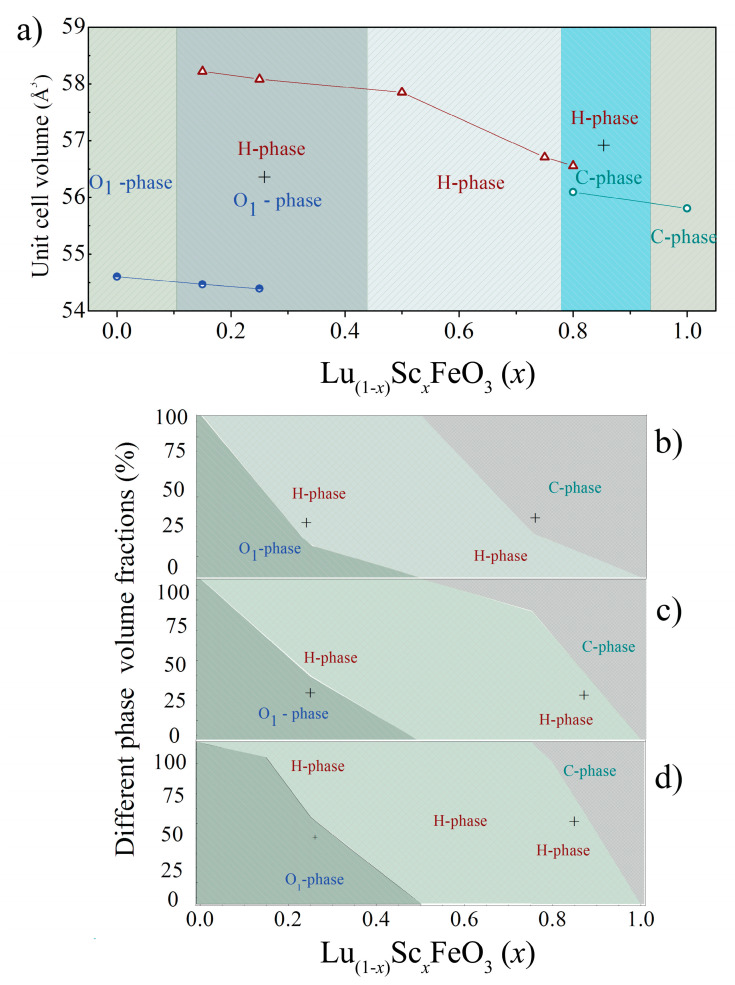
Concentration-driven phase diagram (**a**). Volume fractions of different phases for compounds prepared at high temperatures of 1500 °C—(**b**), 1300 °C—(**c**), 1100 °C—(**d**).

The initial XRD patterns as well as the data refined by the Rietveld method are presented in Figure 1 and Figure S3, which confirm the formation of the solid solutions over the whole concentration range and where several different structural phase regions are observed. As mentioned before, the compound Lu_(1−*x*)_Sc*_x_*FeO_3_ with *x* = 0 is characterized by a single-phase orthorhombic structure (Figure 1); however, an even quite small doping level of 15 mole% already induced notable structural changes. These changes were expressed as the initial formation of an hexagonal phase described by the space group *P6_3_cm* (#185), as indicated by the appearance of the reflections indexed as (110)_H_, (111)_H_ and located around 2*θ*~30.44° and the reflection (002)_H_ around 2*θ*~14.45° (Figure 2). This phase is usually considered quite unstable in the LuFeO_3_ matrix that is most often obtained in thin films because of a high strain occurring between the substrate and the film or in bulk compounds via chemical doping [15,34]. Upon further substitution of lutetium ions by scandium ions up to *x* = 0.50, a gradual increase in the volume fraction of the hexagonal phase was observed as the reflections specific to this phase increased in intensity. Meanwhile, the reflections attributed to the orthorhombic phase became less intensive and, in this case, disappeared completely, resulting in the stabilization of the single-phase hexagonal structure. The single-phase hexagonal structure was also observed for the compound having 75% of Sc content. A further increase of Sc up to 80% led to additional changes and to the appearance of a new phase. This new phase had a bixbyite structure, as shown by the appearance of new reflections indexed as (211)_C_ at around 22.5° and (400)_C_ at around 37.2° (Figure 2). When Lu ions were fully replaced by Sc ions, a single phase with the aforementioned structure was stabilized. Detailed structural data obtained by Rietveld analysis can be seen in Table 1. From these results, one can clearly see that the introduction of Sc^3+^ into the LuFeO_3_ matrix led to a decrease in the volume of orthorhombic lattice, while the unit cell parameters changed in different ways, e.g., the b-parameter firstly increased in the compounds with dopant content up to 15% but then started to decrease. Furthermore, the phase composition showed a gradual onset of the hexagonal phase, while at the initial 15% of scandium content, only a minor amount (9%) of the *P6_3_cm* phase was present. Almost equal phase ratios were measured in the presence of around 25% of Sc dopant; for compounds with *x* = 50% and 75%, the single-phase hexagonal structure was observed. When doping increased up to 80%, a cubic phase with a volume fraction of around 12% formed. The compound with 100% Sc content was single-phase with cubic structure. It is worthy to note that phase stability, as well as particle morphology and size, were notably affected by the synthesis conditions [26,27,35,36]. The structural data are summarized in the form of a phase diagram presented in Figure 3a.

Due to the aforementioned quite intensive background in the ScFeO_3_ sample, it was decided to further anneal the samples containing 0, 0.25, 0.50, 0.75, and 1 Sc at even higher temperatures of 1300 and 1500 °C. The diffraction patterns obtained for these samples can be seen in Figures S4–S8. No significant changes were observed for samples with 0, 0.50, and 1 Sc, besides the narrowing of the reflections, which was most likely caused by an increase of particle size. However, when considering the samples near the phase boundary, viz., those with *x* = 0.25, 0.75, the phase equilibrium seemed to be affected, as a higher synthesis temperature led to a phase transitions from *Pnma* to *P6_3_cm* as well as from *P6_3_cm* to *Ia-3* (Figure 3b–d). For the compound containing 25% of scandium ions, it was noticed that after calcination at 1300 °C, the volume fraction of the hexagonal phase increased from 46% to 60% and finally to 79% when the calcination temperature was 1500 °C. A similar case was observed for the compound with 75% of Sc ions, where the amount of the bixbyite phase increased from 0% at 1100 °C to 20% after calcination at 1300 °C and finally to 80% after sintering at 1500 °C. Overall, these phase transitions indicated that the phase composition and stability were highly affected by the synthesis conditions and could be adjusted accordingly.

### 3.2. Raman Spectroscopy Analysis

**Figure 4:**
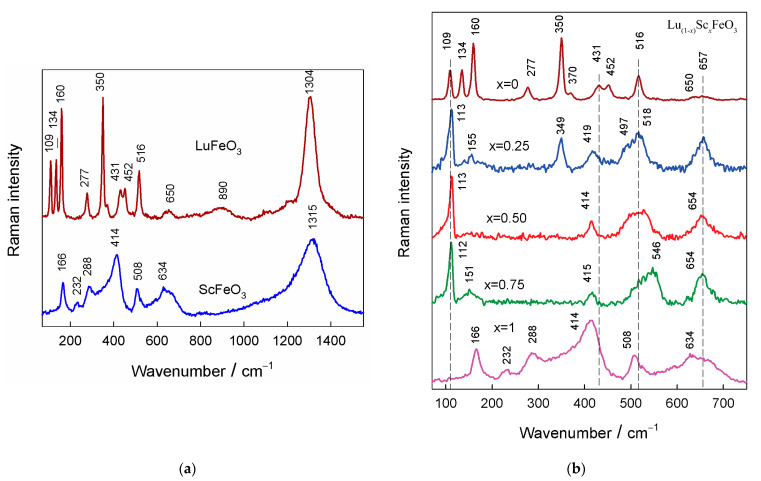
Raman spectra of polycrystalline LuFeO_3_ and ScFeO_3_ (**a**). Composition-dependent Raman spectra of polycrystalline Lu_(1−*x*)_Sc*_x_*FeO_3_ compounds. Intensities are normalized to the intensity of the most intense band, and the spectra are shifted vertically for clarity (**b**).

Raman spectroscopy was employed to access the short-range structural details of the studied compounds. In addition, this technique sensitively probes the presence of defects and disorders [37,38,39]. Figure 4a compares 532 nm excited Raman spectra of polycrystalline LuFeO_3_ and ScFeO_3_ samples sintered at 1100 °C. In the case of orthorhombic orthoferrites with a Pnma space group, one can expect 24 first-order Raman active modes distributed by symmetry in the following way [40,41]:

(1) $${\;\Gamma }_{Raman}=7A_{g}+5B_{1g}+7B_{2g}+5B_{3g}$$

The positions of the fundamental bands were very similar to those in the Raman spectrum of a single-crystal LuFeO_3_ sample [40], thus indicating a high phase purity of the studied compound. Based on the frequency value, the vibrational bands below ~200 cm^−1^ could be considered associated mainly with vibrations of heavy rare-earth ions, the vibrational modes ranging from 200 to 350 cm^−1^ could be related to tilting motions of FeO_6_ octahedra, the bands in the frequency region from 350 to 500 cm^−1^ belonged mainly to the oxygen bending modes, and the vibrational modes at wavenumbers higher than 500 cm^−1^ were related to symmetric stretching vibrations of Fe–O bonds [41,42].

The compound ScFeO_3_ exhibited a completely different spectral pattern (Figure 4a). To the best of our knowledge, there no Raman spectrum of this compound is available in the literature. The XRD measurements indicated the formation of a bixbyite structure having cubic symmetry, described by the space group *Ia-3*. The most intense Raman band for such structure was expected for the *F*g symmetry vibrational mode [43,44]. The corresponding band in the spectrum of the compound was visible near 414 cm^−1^ (*F*_g_). The other same symmetry modes were observed at 288 cm^−1^ (*F*_g_) and 508 cm^−1^ (*F*_g_). The clearly resolved low-frequency band at 166 cm^−1^ probably belonged to the Eg symmetry vibrational mode [43]. The broad band near 634 cm^−1^ had a high contribution from the two-phonon vibrational mode.

Figure 4b shows the composition-induced changes in the Raman spectra of Lu_(1−*x*)_Sc*_x_*FeO_3_ compounds in the frequency region of 70−750 cm^−1^. As discussed above, the spectrum of compound LuFeO_3_ was characteristic of the orthorhombic phase. However, the spectrum of compound with *x* = 0.25 demonstrated prominent structural changes. The intensity of the bands characteristic of the orthorhombic phase near 134 and 160 cm^−1^ considerably decreased, while other lower relative intensity characteristic modes near 277 and 452 cm^−1^ completely disappeared. However, the intensity of the prominent band near 350 cm^−1^ decreased in about times. Importantly, the width of the band determined as full width at half maximum (FWHM) increased from 8.6 to 14.1 cm^−1^. This indicated a considerable distortion of the local structure of the remaining orthorhombic phase. In addition, new intense bands were visible at 113, 419, 497, and 657 cm^−1^. The newly appeared bands were characteristic of the hexagonal phase of LuFeO_3_ and similar compounds [27,45,46,47,48,49]. For the non-centrosymmetric hexagonal crystal symmetry with the P6_3_cm space group, one can expect to observe 38 Raman-active modes distributed by symmetry in the following manner [46,47,49]:

(2) $$\Gamma_{Raman}=9A_{1}+14E_{1}+15E_{2}$$

The most intense band located at 113 cm^−1^ belonged to the *E*_2_ symmetry vibrational mode and can be described as the vibration mode of the heavy Lu ion [47]. The relatively broad band near 419 cm^−1^ (*E*_1_) was attributed to Lu–O stretching vibration, while the shoulder near 497 cm^−1^ (*A*_1_) had a high contribution from the FeO_6_ group bending vibration [47]. Finally, the strong and broad feature at 657 cm^−1^ (*A*_1_) was mainly related to Fe–O stretching motion. Thus, the Raman data indicated the coexistence of the two phases (orthorhombic and hexagonal) for the compound with *x* = 0.25. In addition, the local structure of the orthorhombic phase was highly disordered. The increase in the amount of Sc ions up to *x* = 0.50 resulted in the complete disappearance of the bands characteristic of the orthorhombic phase. This might be related to the transformation of the orthorhombic phase to the hexagonal phase or to the presence of a small amount of highly disordered local structure of the orthorhombic phase. The FWHM value of the band of to the hexagonal phase at 414 cm^−1^ decreased from 24.0 cm^−1^, in the case of the sample with *x* = 0.25, to 15.4 cm^−1^ for the compound with *x* = 0.50, indicating a local structure ordering in the hexagonal phase. Further increase in the amount of Sc ions (*x* = 0.75) resulted only in a perturbation of the complex Raman band near 520 cm^−1^. This band had two components located near 523 and 546 cm^−1^; the higher-frequency blue-shifted component clearly appeared for the compound with *x* = 0.75. Because the vibrational modes in the frequency region above 500 cm^−1^ are related mainly to Fe–O stretching vibrations [41,42], this may indicate a strengthening of the corresponding bond. It should be noted that the spectral parameters of the strong band near 112 cm^−1^ remained essentially unchanged, which suggested that the local structure in the vicinity of heavy Lu ions remained similar.

### 3.3. Scanning Electron Microscopy and EDX Analysis

**Figure 5:**
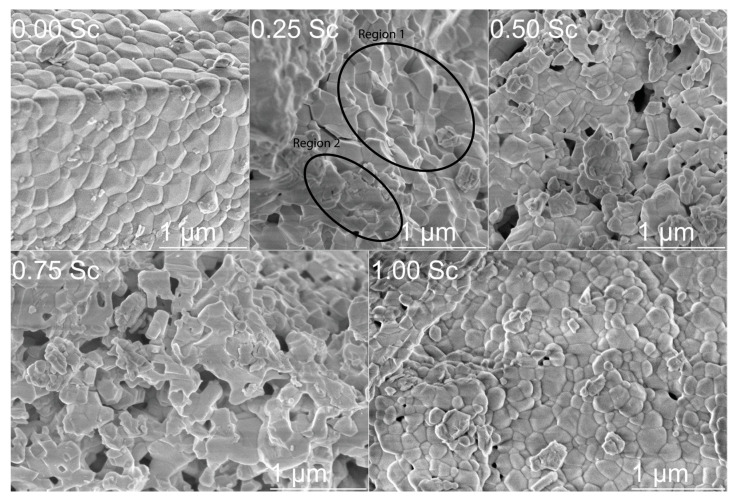
SEM micrographs of Lu_(1−*x*)_Sc*_x_*FeO_3_ (*x* = 0, 0.25, 0.50, 0.75, 1.00) samples.

**Figure 6:**
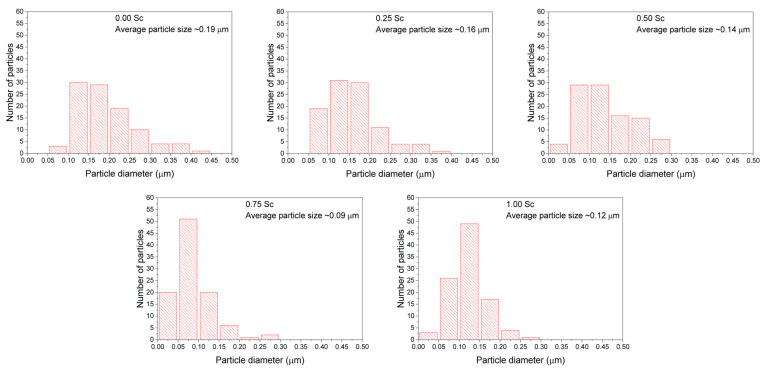
Particle size histograms of Lu_(1−*x*)_Sc*_x_*FeO_3_ (*x* = 0, 0.25, 0.50, 0.75, 1.00) samples.

SEM measurements allowed investigating the effect of Sc doping on crystallite morphology and phase stability (Figure 5). The particle size was measured using ImageJ software and is presented in Figure 6 [50]. The initial compound (*x* = 0) with a single-phase orthorhombic structure was characterized by a rectangular-like shape of the particles, which corresponds to the symmetry of a crystal lattice [51]. The particle size in this compound varied from ~0.05 to 0.45 μm, with an average value of ~0.19 μm. Increasing the scandium content up to 25%, led to significant changes in particle morphology and revealed two distinct regions. One region (region #1) was associated with crystallites of rectangular shape with extremely sharp and discrete edges, while the other region (region #2) was characterized by crystallites with diffuse edges and smeared borders between the individual particles. Furthermore, the average particle size decreased to ~0.16 μm. In the compound with 50% Sc content, the number of particles with diffuse edges was much greater, while the average particle size further decreased to ~0.14 μm. In the compound having 75% Sc content, this trend reached its maximum. SEM data confirmed the model which assumes the formation of particle clusters with no clear borders between each particle, so that the individual particles can scarcely be observed. As such, the average particle size is difficult to determine correctly, and we estimated it to be around ~0.09 μm. The compound without lutetium was characterized by a drastically different morphology of the crystallites, compared to the compounds having a mixed structural state or the hexagonal phase, which mostly resembled the morphology specific for the initial compound LuFeO_3_. The particles had a semi-spherical shape and a particle size in the range from 0.05 to 0.3 μm, with an average size of about 0.12 μm. A broad particle size distribution is quite common for compounds prepared by the sol–gel technique [52,53,54]. Overall, the changes in particle morphology could be related to the change in crystal structure, as the compound with mixed structural state (e.g., *x* = 0.25) or single-phase hexagonal structure (e.g., *x* = 0.50, 0.75) were characterized by a different morphology of the particles. The highest number of particles with no distinct shape and clear borders was most found in compounds with a dominant hexagonal structure. Furthermore, the average particle size decreased with the increase of scandium content, which indicated hampering of the grain growth. This was most likely related to the changes of the melting point of the final compound, as such effects have been previously reported to cause similar changes in particle size [55].

In LuFeO_3_-based compounds, even slight deviations from the intended chemical composition can cause changes in phase transitions and phase stability [56,57]. As such, it is important to further investigate the chemical composition of the samples, and for this reason, EDS measurements were performed. For the initial compound without Sc, the Lu/Fe ratio was measured to be 0.935 (theoretical value was 1), which indicated a homogeneous distribution of the related ions in the sample. In the compound with 25% Sc content, it was observed that the ratio of (Sc + Lu)/Fe was 0.994, also quite close to the theoretical value. Similar results were observed for the other samples as well for that with x = 0.50, where the ratio of (Sc + Lu)/Fe was 0.932, for that with 75% Sc content that had a ratio of 0.914, and for that with 100% Sc, with a ratio of 1.002 (Table 1). While all samples showed a quite homogeneous distribution, the largest difference between the theoretical values was observed for the compounds near the phase boundaries, especially for the compound with 75% Sc content.

Furthermore, from the EDS data, it was determined that the ratio of Sc/(Lu + Sc) was also quite close to the theoretical value for all samples, with a difference of around 3–6%; only for compound having 0.75 of Sc, a higher difference was observed, this potentially caused by inaccuracies during the measurements. Overall, a quite homogeneous distribution of all elements was observed, with no drastic loss or increase in any element. This showed the occurrence of atomic-level mixing of the precursors during the preparation of the compounds using the sol–gel method.

## 4. Conclusions

A sol–gel method was used to synthesize the compounds Lu_(1−*x*)_Sc*_x_*FeO_3_ (0 ≤ *x* ≤ 1) with high phase purity. The compounds with *x* < 0.15 were characterized by a single-phase structural state with orthorhombic structure described by the space group *Pnma*; increase in the concentration of Sc ions led to a structural transition to the hexagonal phase (sp. gr. *P6*_3_*cm*) via a two-phase structural state for the concentration range 0.15 < *x* < 0.45; further chemical doping caused the formation of the single-phase non-perovskite bixbyite structure (sp. gr. *Ia-3*) via two-phase regions, as confirmed by the X-ray diffraction and Raman spectroscopy data. The concentration ranges of the mixed structural states were notably dependent on the synthesis conditions; thus, a high-temperature annealing of the compounds within the morphotropic phase boundary stabilized the phase-specific structure for the heavily doped compounds, viz., the hexagonal phase for the compounds with 0.1 < *x* < 0.4 and the bixbyite structure for the compounds with *x* ≥ 0.8. A strong correlation between the type of structural distortion and the morphology of the crystallites was observed and analyzed, focusing on the compounds with a mixed structural state. Chemical doping also caused a reduction of the average crystalline size from ~0.2 μm for the undoped compounds, down to ~0.1 μm for the heavily doped compounds. Based on the structural data, a preliminary composition-dependent phase diagram was constructed, showing the concentration ranges of the single-phase and the mixed-structural-phase regions.

## Figures and Tables

**Table 1:** Unit cell parameters, reduced volume, and EDX results for Lu_(1−*x*)_Sc*_x_*FeO_3_ (0 ≤ *x* ≤ 1.00) compounds calcinated at 1100 °C.

Sample	Phase	*a*, Å	*b*, Å	*c*, Å	Volume Å ^3^ (Per Reduced Cell)	Sc/(Sc + Lu) Ratio	(Sc + Lu)/Fe Ratio
LuFeO_3_	*Pnma*	5.546(4)	7.557(5)	5.211(3)	54.60(6)	0.000	0.935
Lu_0.85_Sc_0.15_FeO_3_	*Pnma* (91%)	5.533(4)	7.564(2)	5.205(1)	54.46(2)	—	—
	*P6*_3_*cm* (9%)	5.875(8)	5.875(8)	11.688(7)	58.22(5)		
Lu_0.75_Sc_0.25_FeO_3_	*Pnma* (54%)	5.532(1)	7.560(5)	5.201(8)	54.39(2)	0.197	0.994
	*P6*_3_*cm* (46%)	5.869(9)	5.869(9)	11.682(2)	58.08(2)		
Lu_0.50_Sc_0.50_FeO_3_	*P6*_3_ *cm*	5.856(1)	5.856(1)	11.690(1)	57.84(8)	0.447	0.932
Lu_0.25_Sc_0.75_FeO_3_	*P6*_3_ *cm*	5.800(9)	5.800(9)	11.678(8)	56.70(7)	0.713	0.914
Lu_0.20_Sc_0.80_FeO_3_	*P6*_3_*cm* (88%)	5.793(9)	5.793(9)	11.676(1)	56.55(8)	—	—
	*Ia-3* (12%)	9.646(1)	9.646(1)	9.646(1)	56.09(5)		
ScFeO_3_	*Ia-3*	9.629(4)	9.629(4)	9.629(4)	55.80(6)	1.00	1.002

## Data Availability

Not applicable.

## Supplementary Materials

The following supporting information can be downloaded at: https://www.mdpi.com/article/10.3390/ma15031048/s1, Figure S1: XRD patterns of LuFeO_3_ prepared at different sintering temperatures, Figure S2: XRD patterns of ScFeO_3_ prepared at different sintering temperatures, Figure S3: XRD patterns of Lu_(1-*x*)_Sc*_x_*FeO_3_ (0 ≤ *x* ≤ 1), Figure S4: XRD patterns of LuFeO_3_ additionally calcinated at different temperatures, Figure S5: XRD patterns of Lu_0.75_Sc_0.25_FeO_3_ additionally calcinated at different temperatures, Figure S6: XRD patterns of Lu_0.50_Sc_0.50_FeO_3_ additionally calcinated at different temperatures, Figure S7: XRD patterns of Lu_0.25_Sc_0.75_FeO_3_ additionally calcinated at different temperatures, Figure S8: XRD patterns of ScFeO_3_ additionally calcinated at different temperatures.
