# Supplementary material for: Crystal Structure and Concentration-Driven Phase Transitions in Lu(1−x)ScxFeO3 (0 ≤ x ≤ 1) Prepared by the Sol–Gel Method
Source: Materials (Basel). 2022 Jan 29;15(3):1048. doi: 10.3390/ma15031048 (PMC8840425; doi:10.3390/ma15031048)
Supplement: Supplementary file 1 [file materials-15-01048-s001.zip › materials-1552784-supplementary.pdf]

Supplementary data

Crystal structure and concentration driven phase transitions in $Lu_{1-x}Sc_xFeO_3$ ($0 \leq x \leq 1$) prepared by sol-gel method

A. Pakalniškis[1,*], D. O. Alikin[2], A.P. Turygin[2], A.L. Zhaludkevich[3], M.V. Silibin[4,5], D.V. Zhaludkevich[3], G. Niaura[6], A. Zarkov[1], R. Skaudžius[1], D. V. Karpinsky[3,7], A. Kareiva[1]

[1] Institute of Chemistry, Vilnius University, Naugarduko 24, LT-03225 Vilnius, Lithuania
[2] School of Natural Sciences and Mathematics, Ural Federal University, Ekaterinburg, Russia
[3] Scientific-Practical Materials Research Centre of NAS of Belarus, 220072 Minsk, Belarus
[4] National Research University of Electronic Technology "MIET", 124498 Moscow, Russia
[5] Institute for Bionic Technologies and Engineering, I.M. Sechenov First Moscow State Medical University, Moscow 119991, Russia
[6] Department of Organic Chemistry, Center for Physical Sciences and Technology (FTMC), Sauletekio Ave. 3, LT-10257, Vilnius, Lithuania
[7] South Ural State University, av. Lenina, 76, 454080, Chelyabinsk, Russia

Corresponding author: A. Pakalniškis email: andrius.pakalniskis@chgf.vu.lt
Keywords: Phase transitions; structural phase stability; $LuFeO_3$; X-ray diffraction; Solid solutions

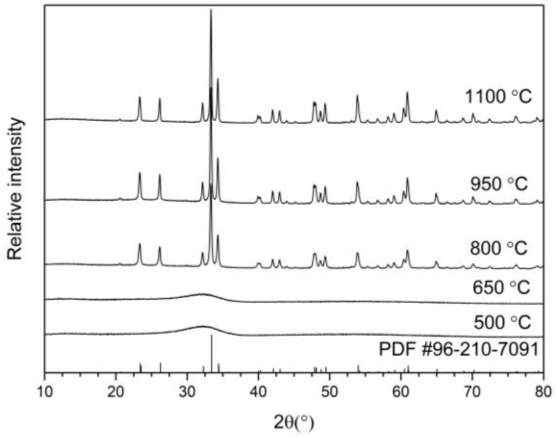

Figure S1. XRD patterns of $LuFeO_3$ prepared at different sintering temperatures

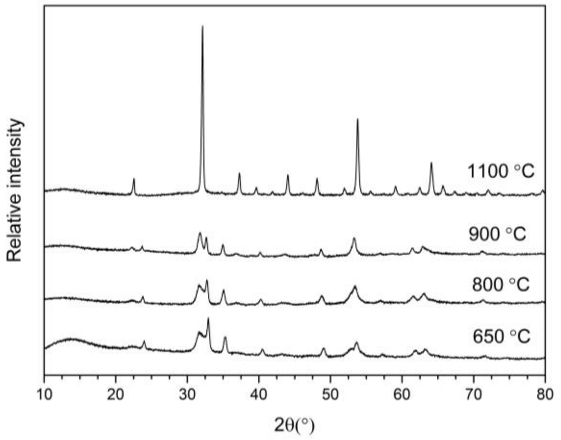

Figure S2. XRD patterns of $ScFeO_3$ prepared at different sintering temperatures

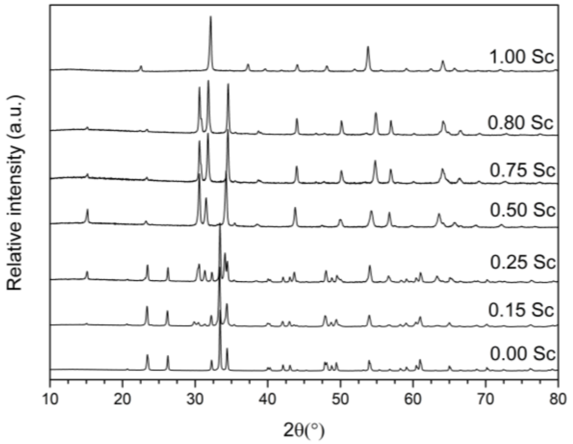

Figure S3. XRD patterns of $Lu_{1-x}Sc_xFeO_3$ ($0 \leq x \leq 1$)

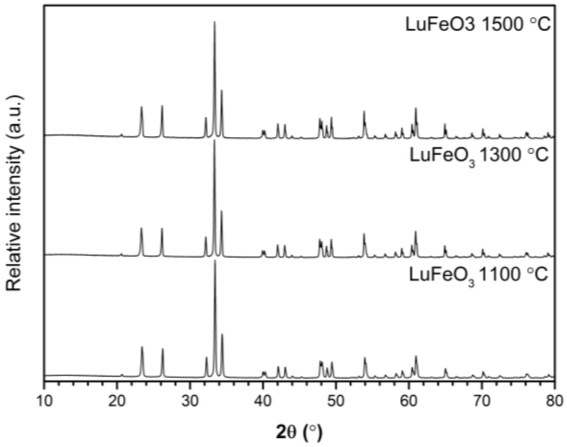

Figure S4. XRD patterns of $LuFeO_3$ additionally calcinated at different temperatures

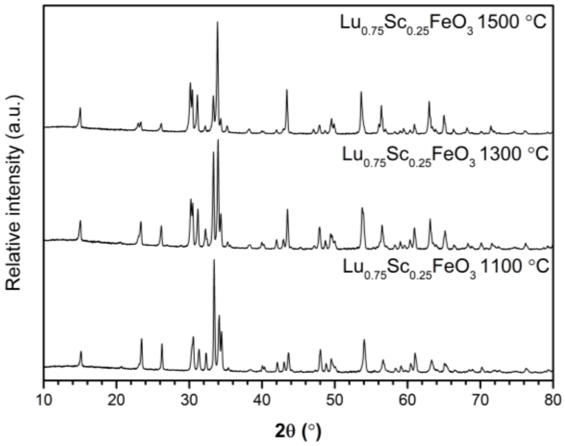

Figure S5. XRD patterns of $Lu_{0.75}Sc_{0.25}FeO_3$ additionally calcinated at different temperatures

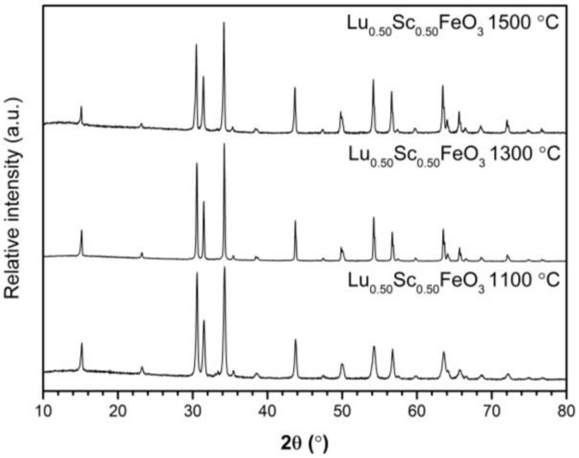

Figure S6. XRD patterns of $Lu_{0.50}Sc_{0.50}FeO_3$ additionally calcinated at different temperatures

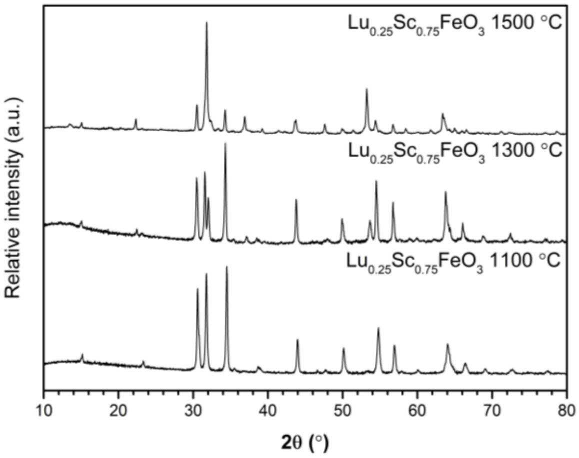

Figure S7. XRD patterns of $Lu_{0.25}Sc_{0.75}FeO_3$ additionally calcinated at different temperatures

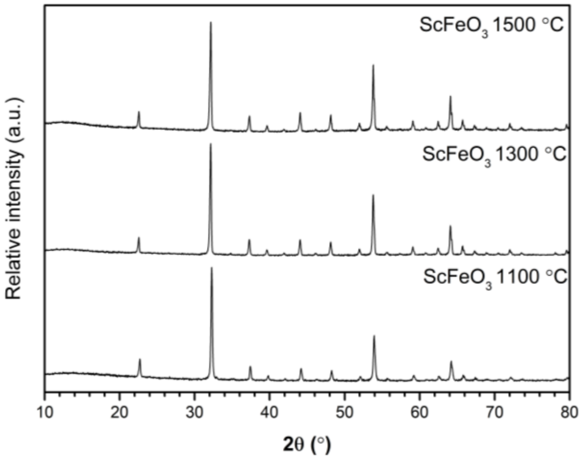

Figure S8. XRD patterns of $ScFeO_3$ additionally calcinated at different temperatures
